# The Economic Evaluation of Mastitis Control Strategies in Holstein-Friesian Dairy Herds

**DOI:** 10.3390/ani13101701

**Published:** 2023-05-20

**Authors:** Melina Richardet, Hernán G. Solari, Victor E. Cabrera, Claudina Vissio, Daniel Agüero, Julián A. Bartolomé, Gabriel A. Bó, Cristina I. Bogni, Alejandro J. Larriestra

**Affiliations:** 1 Facultad de Agronomía y Veterinaria, Universidad Nacional de Río Cuarto, Río Cuarto X5804BYA, Argentina; mrichardet@ayv.unrc.edu.ar (M.R.); alarriestra@ayv.unrc.edu.ar (A.J.L.); 2 Instituto de Física de Buenos Aires-Consejo Nacional de Investigaciones Científicas y Técnicas, Buenos Aires C1428EGA, Argentina; 3 Facultad de Ciencias Exactas, Físicas y Naturales, Universidad de Buenos Aires, Buenos Aires C1428EGA, Argentina; 4 Department of Animal and Dairy Sciences, University of Wisconsin-Madison, Madison, WI 53706, USA; 5 Instituto para el Desarrollo Agroindustrial y de la Salud-Consejo Nacional de Investigaciones Científicas y Técnicas-Universidad Nacional de Río Cuarto, Río Cuarto X5804BYA, Argentina; 6 Facultad de Ciencias Veterinarias, Universidad Nacional de La Pampa, General Pico L6360, Argentina; 7 Instituto Académico Pedagógico de Ciencias Básicas y Aplicadas, Universidad Nacional de Villa María, Villa María X5900, Argentina; 8 Facultad de Ciencias Exactas, Físico-Químicas y Naturales, Universidad Nacional de Río Cuarto, Río Cuarto X5804BYA, Argentina

**Keywords:** *Staphylococcus aureus*, simulation model, segregation, culling, mastitis cost, decision making, total cost

## Abstract

**Simple Summary:**

In order to capture the complexity and dynamics of bovine mastitis, which has multiple effects, such as milk losses, increased risk of culling, or a higher likelihood of reproductive failure, a simulation model was developed. The economic evaluation of the mastitis control was performed considering different interventions combining a basic control plan with either segregation or culling of chronically infected cows. Changes in transmission probability, milk price, and strategies efficacy were evaluated. Several economic parameters in the model could be adjusted to represent farm-specific situations. This is a flexible tool that may support the decision-making of producers and veterinarians.

**Abstract:**

The economic evaluation of mastitis control is challenging. The objective of this study was to perform the economic evaluation of mastitis control, under different intervention scenarios, quantifying the total cost of mastitis caused by *S. aureus* in Holstein cows in Argentina. A model was set for a dairy herd of Holstein cows endemically infected with *S. aureus*. A basic mastitis control plan including proper milking procedures, milking machine test, dry cow therapy, and treatment for clinical mastitis, was compared against other more complex and costly interventions, such as segregation and culling of chronically infected cows. Sensitivity analysis was performed by modifying the intramammary infection transition probabilities, economic parameters, and efficacy of treatment strategies. The basic mastitis control plan showed a median total cost of USD88.6/cow per year, which was close to the infected cows culling scenarios outputs. However, the segregation scenario was the most efficient, in which the total cost was reduced by about 50%. Such cost was more sensitive to probabilities and efficacy than the economic parameters. The model is flexible and can be customized by producers and veterinarians according to different control and herd settings.

## 1. Introduction

Bovine mastitis is the production disease with the greatest economic impact on dairies worldwide [1]. Due to its multifactorial nature, prevalence, and transmission within herds vary greatly depending on the udder health control program carried out by each farm [2]. Both the clinical and subclinical forms of mastitis are responsible for direct costs associated with milk losses [3,4]. Indirect costs involve treatment, veterinary services, reproductive failure, cow culling, and heifer replacement [1]. McInerney et al. [5] considered both types of cost to find ways to minimize the total cost and arrive at an economic optimum for mastitis control [1]. 

Recommended management practices are the basis of adequate mastitis control [6]. These practices include, among others, appropriate management and treatment of clinical mastitis (CM) cases; proper milking procedure; maintenance and adequate use of the milking machine; dry cow therapy; the maintenance of a clean, dry, and comfortable environment for cows; and segregation or culling of chronically infected cows (CIC). In Argentinian dairy farms, CM treatment, post-milking teat disinfection, dry cow therapy, and milking machine testing are the most implemented practices with regard to mastitis [7].

The adoption and consistent implementation of a comprehensive mastitis control program could significantly decrease mastitis losses in dairy farms [7,8]. The lack of adoption of a control program could be due to, in part, the underestimation of the disease losses by the producers [9]. This is especially relevant for those costs that farmers are unable to easily measure, such as reduced milk production and increased culling risk [10]. In addition, an economical approach to value the cost-effectiveness of preventive measures could go a long way toward motivating producers to take up mastitis control programs. 

*Staphylococcus aureus* causes most cases of bovine mastitis in Argentina and many other countries [11,12,13]. Cows get infected with *S. aureus* during milking, and the disease is usually chronic and subclinical with periodic clinical episodes [14]. Segregation and culling CIC are particularly important since these constitute a reservoir of *S. aureus* within the herd [2], which may challenge the long-term effectiveness of the strategies usually followed for contagious pathogens in cows, such as post-milking teat dipping, fore-stripping, and early detection of cases.

An economic evaluation of a mastitis control plan under experimental or observational studies may become a complex, costly, and time-consuming task [15]. In this sense, simulation techniques can be used to quantify the biological and economic consequences of different management strategies [16], such as segregation or culling of CIC. Several models have been developed to evaluate mastitis control in dairy herds in the US [17] and Europe [18,19,20]. Some of them simulate the effects of several pathogen types within different herds [21], and the economic impact of reducing the incidence of CM at the herd level [22]. Others are stochastic and dynamic bioeconomic models that calculate the cost of pathogen-specific intramammary infection (IMI) [18] or assess various intervention strategies for contagious CM by simulating cow- and pathogen-specific IMI transmission [20]. These previous models have been mainly designed to determine the effect mastitis has on the economic performance of dairy farms, but they did not emphasize the economic evaluation of control measures combining different strategies. Furthermore, to the best of our knowledge, no previous models have been proposed that combine a Markov Chain model (to simulate the mastitis evolution in the herd) with a Monte Carlo simulation (to simulate the herd dynamics) under dairy farm conditions in Argentina to study the economics of alternative treatment strategies. Given that *S. aureus* remains the most prevalent major pathogen for dairy herds in Argentina, the present research developed and used a discrete event simulation model to evaluate economically different control scenarios of mastitis caused by *S. aureus* in Holstein cows in Argentina.

## 2. Materials and Methods

### 2.1. Model Framework

**Figure 1:**
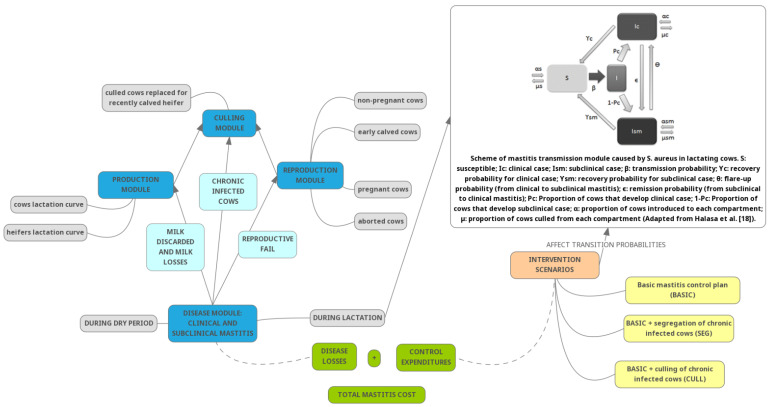
Schematic representation of the simulation model, including modules, intervention scenarios, and components cost.

An individual-based model was built, and events were simulated at the cow level. Each cow was at a given “condition” or state in terms of production (milking or dry cow), reproduction (pregnant or not pregnant), and IMI status (susceptible to infection, presenting CM, or presenting subclinical mastitis (SM)). Culled cows were replaced immediately, and therefore, the number of animals in the herd remained constant. The cows’ condition was updated every two weeks, following a stochastic process that depended exclusively on the collective state of the herd and a transition matrix that incorporated both deterministic and random effects (such as week of pregnancy and contagion, respectively). The transition matrix was sparse and, therefore, better described by those events that produced changes. Strictly speaking, then, we dealt with a Markov model [23]. The complete simulation model was written in C language and is available at: http://200.7.128.5/simulate/TamboWeb/ (accessed on 9 April 2023). A schematic representation of the simulation model is shown in Figure 1. 

First, the model uses information about the status of all the cows through a file that contains their identification, breed, parity, days in milk (DIM), milk production on the last test day, the month of gestation, composite somatic cell count (SCC), and the presence or absence of CM. This file could be provided by users (producers or veterinarians) to apply the model as a farm-specific decision-making tool.

The input parameters about Argentinian economic and production conditions were taken from the literature and consulted with experts. These parameters could be modified by producers or veterinarians according to farm production conditions. 

The model simulated the production-reproduction cycle for each individual cow in the herd along with the transmission of *S. aureus* between cows as the pathogen responsible for infection according to the dataset describing the cows’ initial conditions (Table 1).

The model was subdivided into the following modules.

#### 2.1.1. Reproduction Module

For each cow, the possible reproductive statuses were open (non-pregnant), pregnant, having experienced an abortion, or calving. A voluntary 45-day waiting period after calving was set as a default for each cow, then a 16% pregnancy rate every 21 days (Table 1). The probability of abortion by month of gestation (n = 2 to 8) was set at 7.0, 5.0, 3.0, 1.0, 0.5, 0.2, and 0.2%, respectively, as suggested by Santos et al. [24] and De Vries et al. [25] and adapted to regional data [26].

#### 2.1.2. Production Module

Simulation of the overall lactation output considered the data from the test day provided initially, fitted into average lactation curves for primiparous and multiparous cows using Wood’s model [27] from the Milk Curve Fitter tool from the University of Wisconsin-Madison Dairy Management website [28]. Cows were dried off at the end of the seventh month of pregnancy (Table 1). 

#### 2.1.3. Culling Module 

In this model, different aspects related to culling were set as a default, as detailed below. Every cow that either had an abortion or reached the tenth month of lactation and remained open was subject to culling. Upon reaching their fifth parity, cows could no longer be inseminated and were culled at the end of that lactation (Table 1). The likelihood of death or culling for any other reason (such as diseases other than mastitis) was described by a polynomial function dependent on cow parity and DIM, using estimates for Argentinian dairy herds [26]. Each cow removed from the herd was replaced by a heifer in her last month of pregnancy so that the herd population remained constant over time [20].

**Table 1:** Default input parameters of reproductive, productive, and culling modules of the model.

Parameter	Distribution	Value
Pregnancy probability (per 21 d) ^1^	Discrete	0.16
Probability of abortion by month of gestation (n = 2 to 8) ^1,2,3^	Discrete	3.5, 2.5, 1.5, 0.5, 0.25, 0.1, and 0.1%, respectively
Milk Production (kg) ^3^	Incomplete gamma curve (Wood’s model) ^4^	M_DIM_ = a(DIM^b^)(e^−(c)(DIM)^)—Curve fitting based on herd data input
Pregnancy length (days)	Constant	280
Dry-off probability at 225 days of pregnancy ^5^	Discrete	1.0
Culling probability		
Involuntary ^3^		
First parity	Polynomial	y = 2 × 10^−9^ × DIM^3^ − 3 × 10^−7^ × DIM^2^ − 1 × 10^−5^ × DIM + 0.0112
Second–fourth parity	Polynomial	y = 9 × 10^−10^ × DIM^3^ + 7 × 10^−7^ × DIM^2^ − 0.0002 × DIM + 0.0183
Fifth parity	Polynomial	y = 2 × 10^−10^ × DIM^3^ + 1 × 10^−6^ × DIM^2^ − 0.0003 × DIM + 0.0266
At the end of Fifth parity	Constant	1.0
Non-pregnant with DIM > 300	Constant	1.0
After the abortion	Constant	1.0

M = Milk Yield; DIM = Days in milk; a = Scale factor for initial milk yield; b = Rate factor for the increase in milk yield to peak; c = Rate factor for the decline in milk yield after the peak.^1^ Adapted from De Vries et al. [25]. ^2^ Adapted from Santos et al. [24]. ^3^ Piccardi, 2014 [26]. ^4^ Wood, 1967 [27] ^5^ Kalantari and Cabrera [15].

#### 2.1.4. Disease during Lactation Module

The disease dynamics introduced into the simulation were based on the IMI transmission model described by Halasa et al. [18], which assumes the absence of a latent period or immunity after infection. Thus, cows could eventually fit into three different and mutually exclusive statuses: (1) susceptible to disease (free of IMI), (2) CM infection, or (3) SM infection. The change from one status to another was defined by transition probabilities related to disease transmission, cure (for both CM and SM), from SM to CM (flare-up), and from CM to SM (remission). One of these statuses was randomly assigned at the start of the simulation to each milking cow using regional data on historical prevalence [4,13,29]. This meant that the initial herd values were 88.2% of susceptible cows, 0.3% with CM, and 11.5% with SM.

The IMI dynamics were defined by the parameters in Table 2, briefly described here. The per capita rate at which susceptible individuals were infected was determined as β * I/N [30], where β is the transmission rate, I is the number of infected cows, and N is the total number of milking cows. An infected cow was assigned a certain probability of developing CM (Pc) or SM (1-Pc). If it developed CM, it could recover during the next period (ϒc) or become an SM case (θ). If it had SM during the next period, it could recover (ϒs), become a CM case (ϵ), or remain in the same status (SM). All the average rates were obtained from Swinkels et al. [31] and Halasa et al. [18] and transformed into biweekly probabilities.

SCC levels were stochastically assigned depending on the infection status. Natural logarithm of SCC values (lnSCC) ≤ 3.9 (≤ 50 × 10^3^ cell/mL) was used for healthy cows. The lnSCC values followed a normal distribution (N~5.58; 0.84) in SM cases and a normal distribution (N~6.4; 0.54) in cows with CM [18,32].

#### 2.1.5. Disease during Dry Period Module

The IMI dynamics were modeled separately in dry cows. At the time of dry-off, simulated changes in udder infection status due to *S. aureus* were based on parameters by Halasa et al. [33] under the assumption that all cows were dried off with antibiotic treatment (Table 2).

**Table 2:** Default transition probabilities of *Staphylococcus aureus* udder infection status in milking and dry cows.

Transition Probabilities	Lactation Period Values *			Dry Period Values ^4^
	Default	Lower Limit ^3^	Upper Limit ^3^	
Transmission probability (β)	0.50 ^1^	0.25	0.75	0.007
Recovery probability from clinical IMI (ϒc)	0.35 ^2^	0.14	0.50	0.77
Recovery probability from subclinical IMI (ϒs)	0.10 ^3^	0.06	0.68	0.77
Flare-up probability (from SM to CM, θ)	0.12 ^3^	0.08	0.13	0.09
Remission probability (from CM to SM, ϵ)	1-ϒc			0.22
Probability of becoming a CM case (from IMI to CM, PC)	0.17 ^3^	0.12	0.23	0.10
Probability of becoming an SM case (from IMI to SM, 1-PC)	0.83 ^3^			0.90

* All values were obtained from the literature and converted into biweekly probabilities, the default values used in the model, and lower and upper values used in the sensitivity analysis. ^1^ Adapted from Swinkels et al. [31]. ^2^ With antibiotic treatment of clinical cases, adapted from Halasa et al. [18]. ^3^ Adapted from Halasa et al. [18]. ^4^ Adapted from Halasa et al. [33] for all transition probabilities associated with the dry period.

### 2.2. Effect of Mastitis on Performance

#### 2.2.1. Reproductive Performance

The pregnancy rate decreased when a mastitis event occurred between 2 weeks before and 3 weeks after artificial insemination. Reproductive failure probabilities increased by 50% for cows with CM [34] and 15% for cows with SM [35,36] and were reported as lost reproductive cycles attributed to mastitis.

#### 2.2.2. Milk Production

Losses in milk production attributable to CM were classified into two components. The first involved the milk discarded at the time of diagnosis and that discarded due to antibiotic residues. Losses were deduced considering the lactation curve around the time of the mastitis event and the extent of the treatment. The second component was made up of losses occurring several weeks after the clinical cure. The magnitude of these losses was adapted from Gröhn et al. [37], with the caveat that the cows in our model had 15% less production than those in the reference study, a consideration also made by Halasa et al. [18]. If a cow experienced two or three CM events during the same lactation, discarded milk and production losses were estimated using the values in kg/day of milk reported by Bar et al. [38] (Table 3).

Milk losses due to SM were estimated from the SCC values, using an equation by Dürr et al. [39], which includes a coefficient based on breed, parity, and stage of lactation (BPS). All this information was taken from test day records. The equation was:

DML = (ln(SCC)/1000) − 2) × BPS(1)

where DML is the expected daily milk loss (kg) for a given cow, SCC is the current somatic cell count in that cow’s milk, −2 is the cutoff point in the log scale where losses start, and BPS is the coefficient mentioned before adapted from Dürr et al. [39]. 

If a cow periodically experienced SM after the clinical case, the only losses considered were those due to SM to prevent an overestimation [18].

#### 2.2.3. Culling

Due to the multiple factors involved, the decision to cull a cow is a farm- and context-specific [2]. In the model, it is possible to set a certain number of CM events or SCC peaks above a given threshold within the same lactation to decide whether a cow should be culled [40,41,42].

### 2.3. Strategies and Intervention Scenarios 

Three intervention scenarios were modeled for an Argentinian dairy herd with 200 Holstein cows endemically infected with *S. aureus* whose initial CM and SM daily prevalence were 0.3% and 11.5%, respectively. 

These scenarios were:

(1) Basic mastitis control plan scenario (BASIC), assuming an efficacy to prevent new IMI of 55%. The interventions that were considered in this basic mastitis control plan were post-milking teat disinfection for all lactating cows, fore-stripping, washing and drying teats, and milking machine tests. The efficacies to cure IMI were set at 77% and 35% for dry cow therapy and treatment of clinical mastitis cases during lactation, respectively.
(2) Scenario with segregation (SEG), where the strategies from scenario BASIC were complemented by segregating the CIC. A cow was considered chronic when IMI was detected successively considering two periods of two weeks. These cows were kept separate and milked last.
(3) Scenario with culling, where the strategies from scenario BASIC were combined with the culling of infected cows. Within this scenario, three (3) alternatives were considered: CULL_1_ (cows that had 2 CM in the current lactation); CULL_2_ (cows that had 3 CM in the current lactation); and CULL_3_ (cows that had 6 SCC peaks over 200,000 cells/mL in the current lactation). The culled cows were immediately replaced by a susceptible pregnant heifer in all cases [42].

### 2.4. Economic Calculations

Table 4 lists the initial estimates for the economic parameters measured. The economic impact of CM was estimated as a kilogram of discarded milk multiplied by the market price plus a kilogram of milk yield loss over the lactation curve multiplied by the market price. It was assumed that mastitis milk is not used to feed calves because this could have potentially harmful effects due to endotoxins, and it also would require more intensive management of the feeding program (such as pasteurization due to possible pathogen transmission) with its associated costs. Feeding mastitis milk to calves is not a common practice in Argentina. The economic impact of SM was estimated as a kilogram of milk yield loss multiplied by the market price. For those cows whose milk yield decreased, the potential decrease in dry matter (feed) intake was also considered by subtracting this lower intake expressed as a kilogram of ration multiplied by market price [43]. The economic impact of reproductive losses was estimated as the total number of involuntary days open multiplied by the cost of each day. The cost was calculated considering a second-parity cow between its fourth and fifth month in milk, as described by Cabrera [44]. The cost of the control practices implemented was also calculated for each scenario based on the existing literature and current market prices in US dollars (Table 4). The costs included antibiotics for dry cow therapy and CM treatment, assuming the use of three doses for each episode; teat disinfectant solution, assuming the use of 10 mL per milking cow per day and separate cloths, assuming the use of two cloths per milking cow per day; and the provision of a pen to keep cows segregated. For the maintenance of the milking machine, the cost of three checkings per year was considered. For culling, depreciation at the time of removal of the cow was considered, and the costs of replacement and salvage adjusted for DIM and parity, as described by Pinzón-Sánchez et al. [42]. In all scenarios, the cost of labor was included (Table 4).

### 2.5. Model Run, Sensitivity Analysis, and Model Evaluation

Five hundred iterations were run for a period of 8 yr, with the first 3 yr as burn-in time to reach a steady state. Model outputs were reported as annual median and interquartile range over the last simulated 5 yr period. 

To assess the model´s robustness, a sensitivity analysis was conducted by modifying the IMI transition probabilities, the BASIC scenario efficacy, and the economic parameters. The lower and upper limit values of the transition parameters were taken from the literature and used for this purpose (Table 2 and Table 4). An increase and a decrease of 10% in the preventive efficacy of the BASIC scenario were analyzed. The lower and upper limit values of milk price were taken considering the minimum (0.263) and maximum (0.357) of the historical values of the last five years, representing a variation of around 17% of the average value. Analogously, lower and upper economic values for each practice of the mastitis control plan were defined by decreasing and increasing the default values by 17% (Table 4). In all analyses, changes were made to one parameter at a time while keeping the other values constant.

The sensitivity analysis was used to assess the model’s output under different input values, and since no data from a field trial were available, individual cows were tracked to make sure that the model was logically correct and accurate as needed [45]. The model output for the BASIC scenario was compared against reported data by OCLA [46] regarding the individual cows´ production mean and the proportion of milking cows. Complementary, the number of cases of mastitis estimated by the model was contrasted with the figures reported by Vissio et al. [47]. In addition, veterinarian practitioners (approximately 20 professionals) who attended the 2019 Annual Udder Health Scientific Meeting of APROCAL (Asociación Argentina Pro Calidad de Leche y sus derivados) in Villa María, Córdoba (Argentina) were consulted about the model output. In this meeting, the first author exposed the scopes and assumptions of the model, giving and received feedback about it.

**Table 4:** Default values and ranges were used for the model sensitivity analysis of the economic variables under different intervention scenarios.

Economic Parameters	Default Value	Lower and Upper Values
Average milk price (USD/kg) ^1^	0.308	0.263–0.357
Reproductive cost for involuntary open days (USD/d) ^2^	4.60	-
Feed cost (USD/kg of dry matter) ^3^	0.18	-
Cost of the milking-machine test (USD/yr) ^3^	281.25	233.44–329.06
Average cost of teat disinfectant solution (USD/l) ^3^	2.50	2.08–2.93
Separate cloths (USD/yr) ^3^	620	514.6–725.4
Average cost of dry cow antibiotic (USD/unit) ^3^	1.50	1.25–1.76
Cost of labor (USD/h) ^3^	3.90	3.24–4.56
Average cost of milking cow antibiotic (USD/unit) ^3^	2.00	1.66–2.34
Replacement cost (USD/heifer) ^3^	1300	1079–1521
Culled cow cost (USD/cow) ^3^	600	498–702
Pen cost (USD/yr) ^4^	614	510–718
Labor segregation cost (USD/segregated cow/yr) ^4^	109.50	90.89–128.12

^1^ Observatorio de la Cadena Láctea Argentina (OCLA): annual average of the last five years [48]. ^2^ Cabrera, 2012 [44]. For a second parity cow between the fourth to fifth month in milk. ^3^ Current Market Prices in Argentina. The labor time spent on proper milking procedures, dry cow therapy, and clinical mastitis treatment was 26, 300, and 75 s/cow/each application, respectively [49]. ^4^ Adapted from Huijps et al. [50] for segregated infected cows milked last in a herd size of 200 dairy cows.

## 3. Results

The results were obtained once the model had reached a steady state. In each period (every two weeks), the milking cows´ group reached an average of 87.5% of the herd (q1: 86.5%; q3: 89.0%), with a production average (±SD) of 25.3 ± 1.8 and 27.8 ± 2.1 kg/day of milk for primiparous and multiparous cows, respectively.

As shown in Table 5, the median of CM and SM cases per year in the BASIC scenario was higher than in scenarios SEG and culling (CULL_1_, CULL_2_, and CULL_3_). The BASIC and CULL_2_ scenarios showed higher milk and reproductive cycle losses attributed to mastitis (Table 5). 

**Figure 2:**
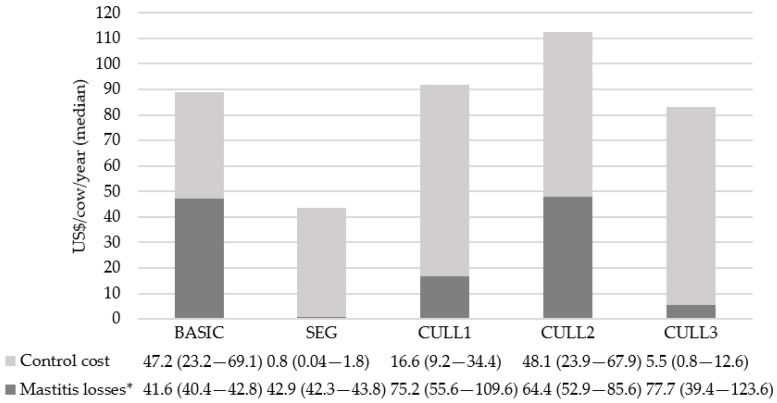
Economic model annual output (and interquartile range in parentheses) for a dairy herd of 200 cows (500 iterations) over a 5 yr simulation period. BASIC: Basic mastitis of prevention and control plan including post-milking teat disinfection for all lactating cows, fore stripping, and washing and drying teats and milking machine test; SEG: the strategies from scenario BASIC were combined with the segregation of CIC kept separated and milking last; CULL: the strategies from scenario BASIC were combined with the culling of CIC (CULL_1_: cows that had 2 CM in current lactation; CULL_2_: cows that had 3 CM in the current lactation; and CULL_3_: cows that had 6 SCC peaks over 200,000 cells/mL in current lactation). * Losses due to milk production and reproductive cycles associated with mastitis cases.

The total cost expressed in USD/cow per year to BASIC, SEG, CULL_1_, CULL_2_ and CULL_3_ scenarios was 88.60 (interquartile range: 63.48–111.98), 43.60 interquartile range: 42.39–45.6), 90.82 (interquartile range: 67.54–144.06), 114.24 (interquartile range: 76.98–151.26), and 84.30 (interquartile range: 41.37–137.31), respectively. The control cost was the most important component within the total cost in SEG and CULL scenarios. Meanwhile, losses due to mastitis and control cost were similar in the BASIC scenario (Figure 2).

The sensitivity analysis showed that out of all the IMI transition probabilities during lactation, transmission probability (β), and recovery probability from subclinical IMI (ϒs) had the greatest influence on the total cost in all scenarios (Table 6). When the efficacy values of the strategies included in the BASIC scenario were set at 49.5%, the total cost increased in all scenarios except in SEG (Table 6). When the efficacy values of the strategies included in the BASIC scenario were set at 60.5%, the total cost decreased by around 80% in all scenarios except SEG, which did not vary (Table 6). The sensitivity analysis in relation to economic parameters associated with the mastitis control plan showed a variation of around 13% in total cost, whereas milk price variation was negligible (Table 4).

The sensitivity analysis allowed checking qualitatively and quantitatively the consistency and credibility of model outputs. In addition, tracking single cows over time was consistent, determining that model’s logic was correct. While the model estimated the level of production as slightly higher than the standard dairy herds in Argentina, the mastitis estimates levels were lower.

Veterinarians attending the most important meeting of practitioners confirmed that the outputs seemed consistent and plausible and, therefore, the model could be used to assess the economic results of different management options for mastitis in dairy herds in Argentina.

## 4. Discussion

The dynamics of *S. aureus* transmission were simulated within a herd to estimate the total cost, disease losses, and control expenditures under different scenarios. Although the main pathogens associated with mastitis are many, this study focused on *S. aureus* because it is the most prevalent among the major pathogens in Argentina [13,29] and worldwide [11,12,51,52] and it is heavily associated with chronic infections [14,53]. The simulation model evaluated the economic results of a basic mastitis control plan without and with either segregation or culling of CIC due to *S. aureus*.

The economic impact of mastitis is strongly linked to its rate of transmission [54]. Our model simulated IMI transmission at the cow level and assessed its economic consequences of several scenarios of udder health control as other models previously developed [18,21,55]. Recently, Gussmann et al. [20] simulated the spread of IMI in a dairy herd at the quarter level, which could be more suitable for quantifying IMI than at the cow level. However, in our model, all the transition probabilities were herd average values regardless of parity or stage of lactation, as described by Halasa et al. [18]. Previous studies have examined different mastitis pathogens [18,20,21], dynamics of infection [20], and units of analysis [20]. The control strategies also differed among studies [20,21]. As a consequence of those differences, comparisons between model outputs are difficult and somehow speculative.

In the BASIC scenario with a basic mastitis control plan, a total of 97.5 cases of *S. aureus*-caused mastitis every 100 cows per year was estimated, and losses due to CM and SM were USD221.0 and USD151.7 kg/case, respectively. The milk losses observed are slightly lower than those reported by Heikkilä et al. [56], which could be explained by the higher level of milk production described in that study. Regarding the reproductive failure losses due to mastitis, the model showed no relevant economic impact in all of the scenarios evaluated. This may be due to the low frequency of cases around the insemination time, where the likelihood of failure is the greatest [34,35,36]. Previous models did not consider reproductive failure within the context of other consequences of mastitis, so we have no data to compare. Under observational settings, Dahl et al. [57] found that the cost of pregnancy losses attributable to CM was around 18% of the total cost of a case, including treatment during the first 75 d of gestation. However, these results are not comparable since we related the reproduction losses to the total cost for cows. In addition to that, the magnitude of the economic impact of the reproduction failure related to mastitis should be thought of as a function of the exposure and frequency.

The total cost approach is based not only on the magnitude of the disease but also on the substitution relationship between losses due to mastitis and expenditures for its control [20,58]. In our study, the total cost of mastitis across different scenarios varied greatly within a range between USD43.6 and USD114.2/cow per year. In other simulation studies with similar intervention strategies, Huijps et al., Halasa et al., and Gussmann et al. [18,20,59] reported values of EUR 49 (USD68), EUR 140 (USD195) and EUR 188 (USD318)/cow per year, respectively. Once again, our focus on the economic impact solely of *S. aureus* may account for a big part of these differences. Furthermore, although Gussmann et al. [20] calculated the total cost at the quarter level, they also considered additional costs, such as an etiological diagnosis by PCR, veterinary visits, and longer treatments, among others that we did not take into account. The scenarios they modeled were not strictly comparable with ours, then, in terms of disease etiology and frequency, control and labor cost, and dairy market price.

According to earlier models, expenses for mastitis control and prevention represent approximately 90% of the total cost [6,18,19,20,21,22,60]; our findings showed similar values when the segregation and culling of cows were taken into account (SEG, CULL_1_, and CULL_3_ scenarios). A previous simulation that included segregation [61] found that the incidence of IMI caused by *S. aureus* decreased, on average, by 35% within 6 to 24 months. In our study, over a 5 years period, the annual IMI incidence was estimated at 1% in the SEG scenario, representing 99% of reduction. The reason for this result can be conjectured in the fact that segregating protects other animals from being infected. The median total cost of the SEG scenario was almost half of the BASIC scenario and was almost completely composed of the control cost, while mastitis losses were negligible. Segregation was always the most cost-effective strategy under all evaluated sensitivity scenarios, so the producers should take this course of action under the model assumptions. The SEG scenario could have additional costs of labor, facilities, and special care that were not accounted for, and therefore, the total cost would have been closer to the BASIC scenario. However, to reach the break-even of both scenarios, the control cost for segregation should be increased by 100%. That would suggest that cost underestimation would not be a critical issue for the SEG scenario to be chosen as a course of action.

Culling CIC has also been shown to prevent mastitis transmission between cows in a herd, especially for contagious pathogens, such as *S. aureus* [2]. However, the rationale behind deciding to cull is complex and usually involves other factors in addition to mastitis [44,62,63]. In the model described here, a new pregnant heifer replaced every cow that was culled; although this may not reflect the real situation within a herd, in other models the same assumption was used [55]. Overall, the inclusion of culling did not modify the total cost due to the increase in expenditures, although milk losses were lower than in the BASIC scenario. Although a recent retrospective study [64] observed a higher total cumulative milk production for cows with mastitis, this effect was not considered in our study because average milk production curves for different lactations were fitted. This would imply an underestimation of the total cost in the CULL_1_ and CULL_2_ scenarios, which were the least profitable anyway. Gussmann et al. [20] also did not consider the level of production of cows with clinical IMI culled and observed only a slightly higher net value. A field trial study in parallel to the simulation model would allow validate the economic results of this study, testing the goodness of estimation of the model compared with real observed values from the field trial. Due to the lack of such field trial data, we evaluated the output model, comparing them with mastitis levels reported in Argentina. The distribution of cases (prevalence and incidence) reported by Vissio et al. [47], based on a sample of 154 herds from Argentina between January 2015 and December 2016, showed values higher than those predicted by the model under the BASIC scenario. Such underestimation may be explained by the fact that other pathogens, in addition to *S. aureus*, may have been present in those herds; moreover, not all herds from Argentina applied a basic mastitis control plan [7]. In addition, we consider the model results are proper for our study purposes based on expert opinions and sensitivity analysis. In general, the model economic output was more sensitive to changes in the transition probabilities than in the economic variables (milk prices and control expenditures) across all scenarios evaluated. Similar observations for transmission coefficients have been made before [18,20,31,54]. In our study, a higher probability of transmission increased the total cost of mastitis, mainly in scenarios CULL_1_ and CULL_3_, caused by the large number of culled cows. This meant a drop in the overall economic efficiency of the control and prevention program. Conversely, when transmission probability decreased, so did mastitis level and associated milk losses, but the expenditures related to the basic mastitis control plan remained similar. In other words, intervention costs cannot go below a certain threshold even at low infection levels; then, the total cost is not greatly affected [20]. The same tendency was observed by changes in recovery and flare-up probability. In the BASIC and CULL scenarios, the sensitivity analysis showed that the total cost was sensitive to efficacy, as expected. This could be explained both by an increase in mastitis losses and control expenditures. However, in the SEG scenario, the total cost was not influenced by most of the parameters considered in the sensitivity analysis, and this could be explained because the segregation of the cows considerably limits the transmission of mastitis and, therefore, the infection levels were kept low without affecting the total cost.

The limitations of this study are related to characteristics of methodology that could influence the interpretation of our results. Therefore, the possibility of extending these results to other conditions is subject to the assumptions of the model. The total cost projections made by our model for mastitis produced by *S. aureus* ignored the effect of other mastitis pathogens interacting at the same time in the herd. This should be considered when assessing the economic results of control strategies. In this sense, our results could be extended to other dairy farms where contagious pathogens, such as *S. aureus*, are the most prevalent. Although the simulation of transmission rates at the quarter level could lead to more realistic IMI estimates [20], our model simulated them at the cow level because the strategies evaluated and the decision-making rested at the cow level. Our model represents a simplification of the biological reality, so the results obtained should be interpreted with caution. Despite this, our model could become a decision support tool that could be used by practitioners at the farm after some training and by those who have the capacity to extract and provide the economics and production conditions of dairy farms. The scenarios described in our study included practically and feasible mastitis management strategies for farmers, so we were able to obtain realistic and applicable results balanced with the theoretical considerations of the simulation. We deemed the strategies and intervention scenarios considered in this study to be the most appropriate according to our expertise and experience. Further studies are necessary to evaluate other alternatives, the effect of the combination of strategies on the total mastitis cost (e.g., segregation followed by culling of CIC), and the inclusion of several pathogens.

## 5. Conclusions

We developed a simulation model to quantify the total cost of mastitis caused by *S. aureus* (milk losses and control expenditures) and to assess the economic trade-offs among different control and prevention scenarios, considering Argentinian market prices and costs. The median total mastitis cost was USD88.6/cow per year under the basic mastitis control plan scenario. In addition, the total cost for scenarios with the culling of CIC showed no relevant differences from the basic plan. In contrast, the total cost, under the SEG scenario, decreased costs by 50%. Under the simulated conditions, the control scenario, including cows’ segregation, was the most efficient. The total cost was more likely to change by manipulating the transition probabilities and efficacy than the economic parameters. Herd disease transmission would remain the driving force for the economic evaluation of mastitis control. The model is a flexible tool that can provide help for mastitis control according to farm production conditions. The model output could support producers´ and veterinarians´ decision-making based on inputs, such as udder health status and economic parameters elucidated from farm records.

## Figures and Tables

**Table 3:** Expected daily milk losses (kg) for clinical mastitis according to parity and a number of occurrences.

	PARITY 1			PARITY ≥ 2		
Relative Time ^1^	First * CM	Second CM **	≥Third CM **	First * CM	Second CM **	≥Third CM **
Same week of CM	−7.12	−5.8	−2.9	−4.65	−5.6	−4.2
+1 wk	−6.78	−4.5	−1.9	−3.10	−5.9	−4.8
+2 wk	−5.41	−2.9	0	−2.81	−3.6	−3.0
+3 wk	−3.71	−2.5	0	−3.05	−2.3	−2.1
+4 wk	−3.00	−2.6	0	−3.25	−1.5	−1.5
+5 wk	−4.58	−2.1	0	−3.67	−0.8	−0.8
+6 wk	−3.81	−1.5	0	−2.70	−0.4	−0.6
+7 wk	−3.11	−1.0	0	−2.70	0.0	−0.1
+8 wk	−2.83	−0.1	0	−1.84	0.0	0.0
+9 wk	−1.56	−0.1	0	−2.29	0.0	0.0
+10 wk and after	−1.52	−0.1	0	−2.29	0.0	0.0

* Adapted from Gröhn et al. [37] for clinical mastitis due to *S. aureus* with a reduction of 15%. ** Adapted from Bar et al. [38]. ^1^ Time of milk measurement about to with concerning to clinical mastitis occurrence.

**Table 5:** Model annual output (median and interquartile range) for a dairy herd of 200 cows (500 iterations) over a 5-yr simulation period.

Model Output		Scenarios			
	BASIC ^1^	SEG ^2^	CULL_1_ ^3^	CULL_2_ ^3^	CULL_3_ ^3^
CM cases/yr	71 (35–101)	1 (0–3)	28 (14–54)	70 (40–97)	6 (1–18)
SM cases/yr	124 (60–177)	1 (0–4)	51 (23–97)	122 (70–171)	14 (2–38)
CM milk losses (kg/yr)	15,689 (7293–21,972)	215 (21–558)	5118 (2782–9708)	17,459 (10,265–22,725)	1610 (256–3924)
SM milk losses (kg/yr)	18,818 (8523–28,173)	398 (0–988)	6070 (3243–12,021)	20,477 (12,591–28,916)	2536 (294–5008)
Reproductive cycle losses attributed to mastitis per year	5 (3–8)	0 (0–0)	2 (1–5)	5 (3–8)	1 (0–2)

^1^ BASIC: Basic mastitis of prevention and control plan including post-milking teat disinfection for all lactating cows, fore-stripping, and washing and drying teats and milking machine test; ^2^ SEG: the strategies from scenario BASIC were combined with the segregation of CIC kept separated and milking last; ^3^ CULL: the strategies from scenario BASIC were combined with the culling of CIC (CULL_1_: cows that had 2 CM in current lactation; CULL_2_: cows that had 3 CM in the current lactation; and CULL_3_: cows that had 6 SCC peaks over 200,000 cells/mL in current lactation).

**Table 6:** The median of mastitis total cost (USD/cow per year) in a herd of 200 dairy cows was obtained from the sensitivity analysis and the relative difference (%) with respect to model annual output (default input parameters).

Parameters	Scenarios				
	BASIC ^3^	SEG ^4^	CULL_1_ ^5^	CULL_2_ ^5^	CULL_3_ ^5^
Default parameters	88.60	43.60	90.82	114.24	84.30
Transmission probability (β)					
0.25	39.77 (↓55.1%)	43.41 (↓0.4%)	39.71 (↓56.3%)	39.76 (↓65.2%)	39.89 (↓52.7%)
0.75	207.84 (↑134.6%)	202.72 (↑365%)	618.27 (↑580.8%)	367.66 (↑221.8%)	1129.49 (↑1239.8%)
Recovery probability from SM ^1^ case (ϒs)					
0.68	39.50 (↓55.4%)	42.52 (↓2.5%)	39.52 (↓56.5%)	39.51 (↓65.4%)	39.52 (↓53.1%)
0.06	159.25 (↑79.7%)	48.12 (↑10.4%)	386.05 (↑325.1%)	277.70 (↑143.1%)	122.71 (↑45.6%)
Recovery probability from CM ^2^ case (ϒc)					
0.14	143.14 (↑61.6%)	63.64 (↑46%)	316.73 (↑248.7%)	204.24 (↑78.8%)	115.82 (↑37.4%)
0.50	52.07 (↓41.2%)	43.40 (↓0.5%)	65.45 (↓27.9%)	58.18 (↓49.1%)	48.82 (↓42.1%)
Flare-up probability (from SM to CM case)					
0.08	104.95 (↑18.5%)	44.66 (↑2.4%)	177.44 (↑95.4%)	122.42 (↑7.2%)	85.50 (↑1.4%)
0.13	71.69 (↓19.1%)	43.71 (↑0.3%)	83.86 (↓7.7%)	108.54 (↓5%)	47.62 (↓43.5%)
Probability of becoming a CM case					
0.12	95.39 (↑7.7%)	44.17 (↑1.3%)	126.05 (↑38.8%)	102.39 (↓10.4%)	96.25 (↑14.2%)
0.23	82.29 (↓7.1%)	43.70 (↑0.2%)	90.80 (↓0%)	99.66 (↓12.8%)	84.88 (↑0.7%)
Basic mastitis control plan cost					
Low-cost ^6^	81.64 (↓7.9%)	36.35 (↓16.6%)	77.92 (↓14.2%)	103.57 (↓9.3%)	70.36 (↓16.5%)
High-cost ^7^	95.74 (↑8.1%)	50.96 (↑16.9%)	104.02 (↑14.5%)	125.42 (↑9.8%)	98.64 (↑17%)
Milk price (USD/kg)					
0.263	86.46 (↓2.4%)	43.60 (↓0%)	93.65 (↑3.1%)	103.64 (↓9.3%)	78.95 (↓6.3%)
0.357	97.02 (↑9.5%)	43.80 (↑0.5%)	108.06 (↑19%)	126.99 (↑11.2%)	86.56 (↑2.7%)
Basic mastitis control plan efficacy					
0.495	145.45 (↑64.2%)	48.63 (↑11.5%)	307.75 (↑238.9%)	203.94 (↑78.5%)	180.39 (↑114%)
0.605	50.77 (↓42.7%)	43.57 (↓0.1%)	55.19 (↓39.2%)	57.26 (↓49.9%)	47.07 (↓44.2%)

^1^ SM: Subclinical mastitis; ^2^ CM: Clinical mastitis; ^3^ BASIC: Basic mastitis of prevention and control plan including post-milking teat disinfection for all lactating cows, fore-stripping, and washing and drying teats and milking machine test; ^4^ SEG: the strategies from scenario BASIC were combined with segregating of CIC kept separated and milking last; ^5^ CULL: the strategies from scenario BASIC were combined with the culling of CIC (CULL_1_: cows that had 2 CM in current lactation; CULL_2_: cows that had 3 CM in the current lactation; and CULL_3_: cows that had 6 SCC peaks over 200,000 cells/mL in current lactation). ^6^ Each practice of the mastitis control plan was reduced by 17%. ^7^ Each practice of the mastitis control plan was increased by 17%.

## Data Availability

The data presented in this study are available in [https://zenodo.org/badge/latestdoi/608841965] (accessed on 9 April 2023).
